# Health Tract, No. 72

**Published:** 1862-06

**Authors:** 


### COFFEE SUBSTITUTES.

THE love of coffee is an acquired taste. Perhaps nine tenths of the families using it "burn" it almost to a coal, so that, in reality, any other burnt bitter would answer quite as well. In fact, multitudes in the far West, removed from markets, have become accustomed to use burnt bread-crust as a substitute, which certainly is not injurious, but it is a known fact that a cup of some mild, hot drink at meals is a positive benefit, while a glass of the purest cold water is as certainly an injury, especially to invalids and to all who do not have robust health.

The following substitutes for coffee have been collected, in all of which it is suggested, first, that the substitute be mixed with the genuine article, half-and-half; second, that in order to know what you are really drinking, roast and grind your own coffee. In this way only can you know that you are not imposed upon, or may not be drinking some cheap material, either filthy or poisonous.

1. It is said that three parts of Rio, with two parts of old government Java, well prepared, is quite as good, if not superior, to that made of the latter alone.

2. WHEAT COFFEE.—Wheat coffee, made of a mixture of eight quarts of wheat to one pound of real coffee, is said to afford a beverage quite as agreeable as the unadulterated Rio, besides being much more wholesome.

3. RYE COFFEE.—Take a peck of rye and cover it with water, let it steep or boil until the grain swells or commences to burst, then drain or dry it. Roast to a deep brown color and prepare as other coffee, allowing twice the time for boiling. Served with boiled milk. Wheat coffee probably could be made the same way.

4. ANOTHER.—Take some rye; first, scald it; second, dry it; third, brown it, and then mix it with one third coffee and two thirds rye, and then you will have as good a cup of coffee as you ever drank.

5. SWEET-POTATO COFFEE.—Take sweet potatoes, cut them fine enough to dry conveniently, and when dried, grind in a coffee-mill; dry them by the fire or stove, at this season of the year, or by the sun, when that will do it; grind and use one and a half tea-cupfuls for six persons, or mixed with coffee in such proportions as you like. Some omit half of the coffee, some more.

6. BARLEY COFFEE.—Take common barley, or the skinless, if it can be obtained, roast as you would coffee, and mix in such proportion as suits your taste. It is very good.

7. PEA COFFEE.—It is probably known to many that a very large per cent of the ground coffee sold at the stores is common field-peas, roasted and ground with the coffee. There are hundreds of thousands of bushels of peas annually used for that purpose. Those who are in the habit of purchasing ground coffee can do better to buy their own peas, burn and grind them, and mix to suit themselves.

8. CARROT COFFEE.—Is recommended by an exchange. Cut up, dry and grind, and mix with coffee in quantities to suit the taste.

9. CHESTNUT COFFEE.—Chestnuts, also, are said to make excellent coffee.

10. Dandelion root, dried and slightly scorched, never burned.

11. CHICORY COFFEE.—Equal weights of chicory and coffee, dried and roasted in the usual manner. The chicory root is raised as easily as carrots, and in exactly the same manner. To prepare the root, wash it clean, slice lengthwise in four to six pieces, according to size, cut in two-inch lengths, dry and keep in a dry place until wanted. Chicory is largely used to adulterate coffee in this country, and especially in Europe, 25 million pounds being used in England and France alone.

12. EXCELSIOR COFFEE, (our own.)—Half a cup of pure, new, farm-house milk, (such as is furnished to New-Yorkers by the Rockland County and New-Jersey Milk Association,) and while almost boiling hot, add to it as much boiling water, and when sweetened to suit, call it "coffee," and drink it down.

It is worthy of remark, that if the same preparation be provided for children for supper, and you simply call it "tea," they would not perceive any difference between it and the coffee for breakfast. After several years use of both, we have never been able ourselves to perceive the slightest difference.

